# Public health education at China’s higher education institutions: a time-series analysis from 1998 to 2012

**DOI:** 10.1186/s12889-018-5605-4

**Published:** 2018-05-31

**Authors:** Jianlin Hou, Zhifeng Wang, Xiaoyun Liu, Youhui Luo, Sabhyta Sabharwal, Nan Wang, Qingyue Meng

**Affiliations:** 1 Institute of Medical Education & National Center of Health Professions Education Development, Peking University, Beijing, 100083 China; 2 School of Public Health, Peking University, Beijing, 100083 China; 3 China Center for Health Development Studies, Peking University, Beijing, 100083 China; 4 Office of Education, Peking University Health Science Center, Beijing, 100083 China; 5 University of Hawaii-Mānoa, Honolulu, HI 96822-2319 USA; 6 Jishuitan Hospital, Beijing, 100035 China; 7 School of Public Health & China Center for Health Development Studies, Peking University, Mailbox 505, 38 Xueyuan Road, Haidian, Beijing, 100083 China

**Keywords:** China, Public health, Education, Scale of education, Structure of education, Human resources for health

## Abstract

**Background:**

Although China’s modern education for public health was developing over the past 60 years, there is a lack of authoritative statistics and analyses on the nation’s development of education for public health at higher education institutions (HEIs). Few quantitative studies on this topic have been published in domestic and international peer-reviewed journals. To address this knowledge gap, we aimed to use national data to quantitatively analyse the scale, structure, and changes of public health education in China’s HEIs, and to compare the changes of public health education with those of other health science disciplines.

**Methods:**

This study uses previously unreleased national data provided by the Ministry of Education of China that includes the number of health professional students by school and major. The data, which spans from 1998 to 2012, are descriptively analyzed.

**Results:**

The number of HEIs for public health education per 100 million population increased from 7.2 in 1998 to 11.3 in 2012. The total enrolment, number of students, and number of graduates increased at rates of 7.3, 7.4, and 5.8% per year, respectively. The percentage of junior college students dropped drastically from 24.0 to 8.4% from 1998 to 2012. During that same period, the number of undergraduates, master and doctorate students increased. Undergraduates accounted for the majority of public health graduates (63.1%) in 2012, and master and doctorate students increased by 10.0 and 5.1 times, respectively, from 1998 to 2012. The relative percentage of public health enrollment, students, and graduates to all health education disciplines dropped from about 6.0% percent in 1998 to around 2% in 2012.

**Conclusions:**

The overall scale of public health education has clearly expanded, though at a slower pace than many other health science disciplines in China. The increase of public health graduates helped to address the previous shortage of public health professionals. Gradually adopting a modern model of education, public health education in China has undergone notable changes that may be informative to other developing countries though it still faces a complex situation in terms of graduates’ adherence to public health, student recruitment, teaching and training, program planning and reform.

## Background

Socioeconomic development and changes in lifestyle behaviors lead to increasing demands for preventive medicine and public health services. As the World Health Organization pointed out in its report, ‘Life in the 21^st^ century: a vision for all’, there is a shift to focus on preventive medicine in effort to improve and maintain baseline health [1]. In China, non-infectious chronic diseases (NCDs) have become a serious health threat, and are recognized as a severe public health problem. National data showed that one in five people in China have been diagnosed with at least one chronic disease, and deaths caused by chronic diseases comprise an estimated 80% of total mortality [2, 3]. This chronic disease burden constituted 70% of total disease burden in 2005 [2, 3]. While the incidence rates of infectious diseases dropped drastically in recent years, infectious diseases continue to be major threats to the health of the Chinese people [4]. The outbreaks and onslaught of Severe Acute Respiratory Syndrome (SARS), avian flu, and HIV/AIDS provides evidence that the threat of infectious disease is a prevalent issue. Also, aside from challenges from both chronic and infectious diseases, China faces other public health issues such as a rapid aging population, smoking, environment pollution, obesity, and other health disorders [5–7]. In order to meet these challenges, competent public health professionals are urgently needed by the Chinese society. However, there is still a shortage of public health professionals in China [8, 9]. For example, the number of health professionals for disease prevention and control is around 14 per 100,000 people in China, which is only one-fifth of that of the United States [10]. The number of health professionals at China’s Centers for Disease Prevention and Control (CDCs) decreased from 157,000 in 2005 to 147,000 in 2011 [11]. Nearly half of China’s public health professionals have graduated from secondary schools with a vocational degree (i.e. *Zhong Zhua*n), adding to concerns regarding the public health professionals’ level of education [8]. Therefore, China’s public health education system is faced with the task of coping with increasing demands for its public health workforce.

As the most populous country and largest producer of health professionals in the world, China has a vast and complex system of health professional education [3]. Because the Chinese government adopted a prevention-first policy to guide its health efforts, public health has been an indispensable component of health professional education in the country. When the People’s Republic of China was founded in 1949, public health, formerly called “hygiene”, was one of the only four undergraduate majors for health professionals, and remained as a core major though the official list of majors was revised several times. Since then, public health and preventative medicine have been first-level disciplines in education of health professionals.

The scope of education for public health has continued to evolve and expand in China. From the 1950s to 1970s, the Chinese model of public health education was adopted from the former Soviet Union, placing emphasis on sanitation and hygiene. During this era, many medical universities and colleges established departments of hygiene or, as it was later called, preventive medicine. Public health education mainly consisted of five core disciplines: food and nutrition, environment health, labor health, radiation health, and school health [10, 12, 13]. Beginning in the 1980s, departments of preventative medicine began founding schools of public health [14]. New disciplines were also established, based on western models, which included epidemiology, health statistics, social medicine, and health policy and management. In 2002 and 2003, the outbreak and spread of SARS not only raised people’s attention to public health and infectious diseases prevention, but also led to a strong call for the education and training of health professionals to tackle public health emergencies [15]. In the past few years, globalization and the aging population triggered the establishment of some new disciplines, including global health and elderly health [16].

Sorted by levels of education attainment, public health education programs at China’s higher education institutions (HEIs) can be classified into four types: 3-year junior college (i.e. *Da Zhuan*), 4- or 5-year undergraduate/bachelor, 3-year master, and 3-year doctorate programs. Master and doctorate education belongs to graduate education, of which the focus has shifted from academic education (e.g. PhD degree programs) to professional education (e.g. Master of Public Health degree programs) [10]. A number of the most competitive universities also offer 7-year combined undergraduate-master programs, and 5-year combined master-doctorate programs. The Master of Public Health (MPH) program was initialized in 2002 [17]. As of 2010, there were 24 universities that were authorized to admit MPH students, with an annual enrollment of about 1500. The annual enrollment of undergraduate, master, and doctorate public health students were about 7000, 1500, and 300 to 400, respectively [18].

China’s public health workforce exceeds 0.8 million people [19]. Modern education for public health has been developing for more than 60 years in the country. Nationally, China is estimated to have the largest number of public health schools in the world, totaling 72 in 2008 [20]. To the best of our knowledge, there is a lack of authoritative statistics and analyses on the nationwide development of education for public health. Only a few quantitative studies on this topic were published in both domestic and international peer-reviewed journals. To address this knowledge gap, we aimed to use national data to conduct a quantitative analysis of the scale and structure of education for public health at China’s HEIs and their changes. By connecting the results of data analysis with existing research and the most recent Chinese health-professional education reform, we also discuss policy suggestions and implications for the reform of public health education in China and other developing countries.

For China’s higher education, the year 1998 was important because several educational policies were implemented by the central government around this year, which posed lasting and varied effects on education of health professionals. These policies include numeric expansion of enrollment, further development of private education institutions, and university mergers [3]. Therefore, our research question in this paper focused on: 1) how the overall scale and structure of public health education had changed since 1998, and 2) how public health education had performed when compared with other health science disciplines from 1998 to 2012.

## Methods

Data used in this study are previously unreleased national data provided by the Ministry of Education (MOE) of China, which include the numbers of health professional students by school and major. The datasets were generated from yearly statistical forms that each higher education institution submitted to local education authorities and the MOE [3]. The data, spanning from 1998 to 2012, were analyzed by descriptive analysis.

Our analysis of the MOE dataset was focused on higher education institutions (HEIs) that offer education for public health, with at least one program in the first-level discipline: public health and preventive medicine. These programs are offered through junior college, bachelor, master, or doctorate degrees.

China’s HEIs can be grouped into two types: regular HEIs and HEIs for adults. Typically, the former admits high-school graduates as full-time students by the national college entrance examination, while the latter provides adults with higher education on a part-time basis through distance education or select on-campus courses. Regular HEIs may best represent China’s higher education of health professionals because they are dominant in the educational system and all top HEIs are regular ones. Furthermore, a person who wishes to study health science at HEIs for adults should be an in-service health professional or have obtained a license for practice [21], making it difficult to estimate the effects of education output on the development of health workforce. Therefore, only regular HEIs are included in our analysis.

## Results

### Overall scale and its changes

**Fig. 1:**
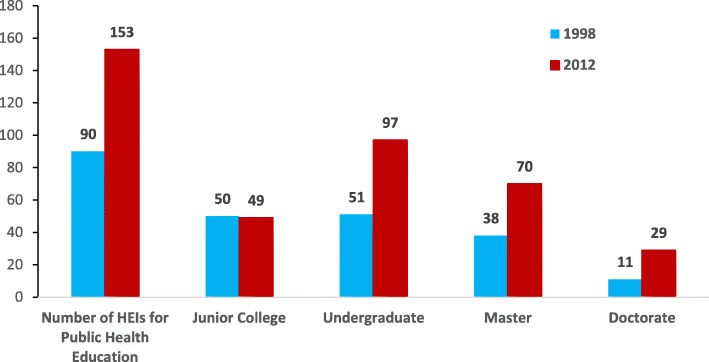
Number of higher education institutions that offered education for public health

As shown in Fig. 1, the number of HEIs offering public health education programs has increased from 90 to 153 institutions or from 7.2 per 100 million population to 11.3 per 100 million population between 1998 and 2012. The number of HEIs providing junior college programs decreased slightly, from 50 programs in 1998 to 49 programs in 2012. By contrast, HEIs with doctorate-level public health education programs more than doubled during the same timeframe. HEIs with either undergraduate or master programs totaled 97 and 70 in 2012, and both programs increased enrollment by more than 80% since 1998.

**Fig. 2:**
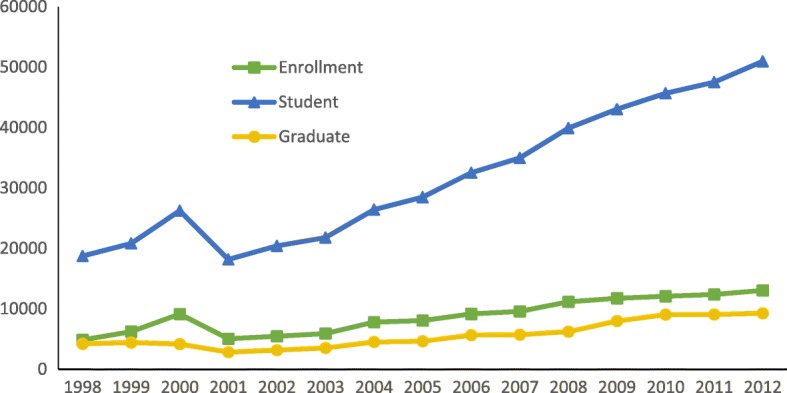
Number of public health education enrollment, students and graduates: 1998–2012

During 1998 to 2012, education for public health was averaging an annual growth rate of 7.3% for enrollment, 7.4% for the number of students, and 5.8% for the number of graduates. In other words, the number of public health enrollment, students, and graduates increased by 2.7, 2.7, and 2.2 times, respectively (Table 1, Fig. 2). However, when compared to other health professional education, the relative percentage of public health education decreased, indicating a greater increase in enrollment for other health disciplines than public health. Specifically, the relative percentage of public health enrollment, students, and graduates to all health education disciplines dropped from about 6.0% percent in 1998 to around 2% in 2012 (Table 1).

**Table 1:** Number and relative percentage of public health education enrollment, students and graduates

Year	Enrollment		Student		Graduate	
	N	%^a^	N	%^a^	N	%^a^
1998	4854	5.9	18,739	6.2	4209	6.4
1999	6221	5.3	20,808	5.9	4437	6.7
2000	9138	5.7	26,237	5.8	4181	6.4
2001	5052	2.7	18,167	3.2	2853	4.1
2002	5462	2.5	20,419	2.9	3183	3.7
2003	5912	2.1	21,801	2.5	3537	2.9
2004	7808	2.4	26,413	2.6	4530	2.7
2005	8061	2.2	28,481	2.4	4656	2.2
2006	9158	2.2	32,518	2.4	5666	2.1
2007	9572	2.4	34,972	2.4	5734	1.8
2008	11,157	2.6	39,906	2.5	6231	1.6
2009	11,743	2.4	43,040	2.5	7995	1.9
2010	12,079	2.3	45,662	2.5	9058	1.9
2011	12,374	2.1	47,500	2.4	9090	1.9
2012	13,048	2.2	50,949	2.4	9268	1.8

^a^Relative percentages compared to total number of enrollees, students, and graduates for all health-related disciplines

**Fig. 3:**
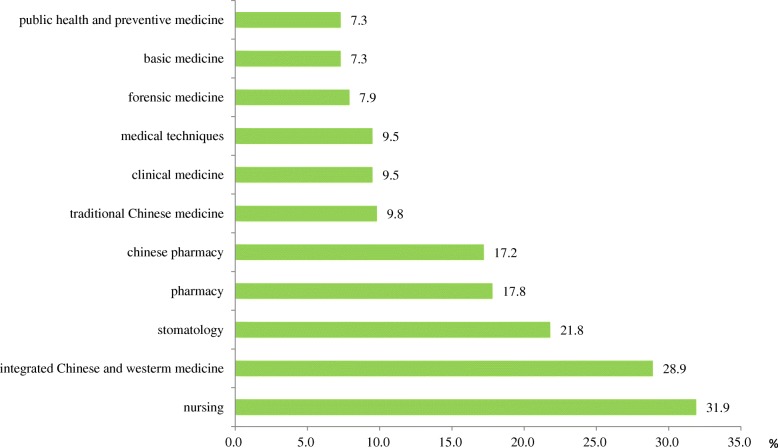
Average growth rate of enrollment: 1998–2012

As shown in Fig. 3, public health enrollment increased at a rate of 7.3% per year, which was much lower than the other health-related disciplines such as nursing (31.9%), integrated Chinese western medicine (28.9%), stomatology (21.8%), and pharmacy (17.8%).

### Structure of education

The number of public health undergraduate, master, and doctorate students increased from 1998 to 2012, with an average growth rate of 8.0, 16.2, and 15.9%, respectively. Meanwhile, the percentage of junior college students decreased from 24.0 to 8.4%. Undergraduate students consistently outnumbered the other types of students, accounting for about 70% of public health students in these years (Table 2).

**Table 2:** Number of public health students by educational levels: 1998–2012

Year	Junior college	Undergraduate	Master	Doctorate	Total
1998	4503(24.0)	13,334(71.2)	754(4.0)	148(0.8)	18,739(100.0)
1999	4676(22.5)	15,059(72.4)	891(4.3)	182(0.9)	20,808(100.0)
2000	6674(25.4)	18,165(69.2)	1158(4.4)	240(0.9)	26,237(100.0)
2001	2362(13.0)	14,357(79.0)	1147(6.3)	301(1.7)	18,167(100.0)
2002	2686(13.2)	15,900(77.9)	1485(7.3)	348(1.7)	20,419(100.0)
2003	2851(13.1)	16,684(76.5)	1827(8.4)	439(2.0)	21,801(100.0)
2004	4078(15.4)	19,584(74.1)	2209(8.4)	542(2.1)	26,413(100.0)
2005	3119(11.0)	22,000(77.2)	2687(9.4)	675(2.4)	28,481(100.0)
2006	3685(11.3)	24,879(76.5)	3181(9.8)	773(2.4)	32,518(100.0)
2007	3963(11.3)	26,432(75.6)	3735(10.7)	842(2.4)	34,972(100.0)
2008	4967(12.4)	29,652(74.3)	4319(10.8)	968(2.4)	39,906(100.0)
2009	4783(11.1)	32,419(75.3)	4885(11.3)	953(2.2)	43,040(100.0)
2010	4474(9.8)	34,698(76.0)	5406(11.8)	1084(2.4)	45,662(100.0)
2011	4111(8.7)	36,240(76.3)	6057(12.8)	1092(2.3)	47,500(100.0)
2012	4277(8.4)	39,362(77.3)	6144(12.1)	1166(2.3)	50,949(100.0)

Percentage in parenthesis

In 1998, junior college graduates accounted for nearly half (46.4%) of public health graduates and the percentage dropped to 14.4% in 2012. Undergraduate graduates accounted for the majority of graduates with 63.1% in 2012, while master and doctorate graduates respectively increased by 10.0 and 5.1 times from 1998to 2012 (Table 3).

**Table 3:** Number of public health graduates by educational levels: 1998–2012

Year	Junior college	Undergraduate	Master	Doctorate	Total
1998	1954(46.4)	2024(48.1)	184(4.4)	47(1.1)	4209(100.0)
1999	1889(42.6)	2293(51.7)	218(4.9)	37(0.8)	4437(100.0)
2000	1597(38.2)	2321(55.5)	231(5.5)	32(0.8)	4181(100.0)
2001	620(21.7)	1944(68.1)	225(7.9)	64(2.2)	2853(100.0)
2002	476(15.0)	2364(74.3)	289(9.1)	54(1.7)	3183(100.0)
2003	749(21.2)	2308(65.3)	397(11.2)	83(2.3)	3537(100.0)
2004	968(21.4)	2964(65.4)	482(10.6)	116(2.6)	4530(100.0)
2005	760(16.3)	3198(68.7)	568(12.2)	130(2.8)	4656(100.0)
2006	1280(22.6)	3536(62.4)	692(12.2)	158(2.8)	5666(100.0)
2007	1037(18.1)	3618(63.1)	902(15.7)	177(3.1)	5734(100.0)
2008	1394(22.4)	3654(58.6)	1012(16.2)	171(2.7)	6231(100.0)
2009	1400(17.5)	5094(63.7)	1293(16.2)	208(2.6)	7995(100.0)
2010	1743(19.2)	5689(62.8)	1402(15.5)	224(2.5)	9058(100.0)
2011	1315(14.5)	6010(66.1)	1501(16.5)	264(2.9)	9090(100.0)
2012	1331(14.4)	5848(63.1)	1848(19.9)	241(2.6)	9268(100.0)

Percentage in parenthesis

## Discussion

This study provides important information regarding the overall scale and changes of the public health education at China’s HEIs between 1998 and 2012. We found that the number of public health education institutions, enrollment, students, and graduates generally increased over the years. The expansion of public health education has played a positive role in addressing the deficit of the public health workforce. Indeed, the number of public health workers in China increased from 533,000 in 2005 to 667,000 in 2012 [22]. From this study, we can also support that the decrease in junior college enrollment and increased enrollment in undergraduate, master, and doctoral programs directly affected the education attainment composition of public health professionals. By 2012, 28.7% of public health practitioners had a higher educational attainment beyond undergraduate level [23]. In China’s CDCs, this percentage grew from 15.5% in 2005 to 30.7% in 2010 [11].

But, while the overall scale of public health education expanded, it occurred at a slower pace than many other health science disciplines. At one of the most competitive universities in China, doctorate students admitted into public health and preventive medicine programs only increased 5.3% from 2008 to 2011, which was substantially lower than the average 14.1% increase for all disciplines within the university. Also, the enrollment quota at the university was considered to be insufficient for the public health and preventive medicine programs [24]. Although China plans to increase the public health workforce to 1.2 million by 2020, the country has yet to establish a national-level plan to regulate the increased scale of public health education [22]. Several important issues, such as the gap between supply and demand for the public health workforce, are still waiting to be well studied. Another remaining question concerns how best to increase the overall scale of public health education.

A majority of public health graduates are employed by CDCs, institutes of health inspection, or hospitals [25, 26]. Nevertheless, some scholars believed there might be an oversupply of public health graduates since many public health graduates also commonly found jobs outside the healthcare system [27]. The School of Public Health (SPH) of Fudan University, widely known as one of the best SPHs in China, reported that nearly one third of its undergraduate graduates in 2008 were employed by enterprises that were unrelated to any type of health field or science [28]. The most frequently cited factors for this phenomenon were associated with remuneration, geographic locations, and career advancement [28, 29]. According to a later study on the same school, some master and doctorate graduates also were employed by enterprises. Between 2009 and 2013, the proportion of finding a job at enterprises ranged from 11.8 to 20.5% for master graduates and 0 to 19.4% for doctorate graduates [30].

Another issue was that many students were often persuaded or assigned to study public health. According to a nationwide survey of 1197 public health undergraduate students, those who chose to study public health as their first preference only accounted for 26.9% of the entire surveyed students, which starkly contrast against the preferences for students who chose to study medicine (75.0%). Due to a large proportion of students that were dissatisfied with their majors, these students were more likely to find a job that is not related to health science. The survey also found that about one in five public health student were unwilling to find a job in public health after graduation [31]. Furthermore, the education for public health students in China lags behind due to outdated curriculum and teaching materials [18], excessive focus on teaching biomedical sciences [8], insufficient or low quality practical training in the public health sector [6, 32]. Finally, although it is essential to equip future physicians with the knowledge of disease prevention and support their ability to deal with public health emergencies [33], insufficient attention was given to teaching public health and building public health competencies in the medical education in China, leaving medical students with poor awareness of disease prevention and creating a deficiency in training for these students on infectious diseases and population health topics [34, 35].

Therefore, education for public health in China faces a complex situation, with much work still be done, especially with the introduction of a new national health-professional education reform. In 2017, the State Council called for a close correlation between the education of health professionals and Chinese health system’s demand for health professionals. This emphasizes a major aim of the education reform: to increase the supply of those health professionals in highest demand, and to improve the quality of those graduates. A number of measures were suggested to strengthen the reform: establishing a balancing mechanism between the supply and demand of health professionals to optimize the scale and structure of education, prioritizing standardized education to improve the quality of graduates, and improving incentive policies for health professionals. In the first health professional education policy issued by the General Office of the State Council since the founding of the People’s Republic of China in 1949, public health professionals are listed as one of the health professionals that are scarce and urgently needed by the Chinese society, indicating a need to continue expanding the overall scale of public health education [36]. In the near future, concrete actions may be needed to accelerate the reform progress in education for public health. To optimize the overall scale of education, it may be necessary to plan and reform public health education by developing evidence-based research and policy-making. Meanwhile, it is also important to take measures to recruit students who are more likely to stay in public health. In addition to improving the public health curriculum, China’s HEIs may consider providing MD (Doctor of Medicine)- MPH (Master of Public Health) dual degree programs.

Considering the magnitude of China’s public health workforce and education, the country has great potential to serve as a role model for other developing countries in public health education and regulation. Gradually adopting a modern model of education, public health education in China has undergone notable changes that may be informative to other developing countries. Changes include expanding scale to address the shortage of public health workers, adjusting educational levels to improve education attainment composition, and diversifying disciplines and programs to support specialized public health professionals. With the implementation of the new reform and its continuous efforts to improve the supply of the public health workforce, China is capable of continuing to contribute significantly to the international arena of public health education.

## Conclusions

While the number of public health students at HEIs has been growing over the past couple of years and could potentially begin to address China’s need for public health professionals, it is expanding at a slower pace when compared to other health science disciplines. Also, the lack of commitment to public health agencies after graduation might continue to create gaps in the public health workforce.

Generally, it may be necessary to plan and reform the enrollment of public health education programs in order to optimize the overall scale of education, as well as recruit students who are more likely to stay in public health. Incentives should also be provided to encourage graduates to work in public health institutions. Furthermore, integration of public health competencies into medical training are strongly recommended.

## Abbreviations

CDC: Center for disease prevention and control
HEI: Higher education institution
MD: Doctor of medicine
MOE: Ministry of Education
MPH: Master of public health
NCD: Non-infectious chronic disease
SARS: Severe acute respiratory syndrome
SPH: School of Public Health
